# The role of *LARP1* in breast cancer progression: from prognosis to immune microenvironment remodeling

**DOI:** 10.3389/fendo.2026.1764944

**Published:** 2026-06-16

**Authors:** Yangtian Ye, Hong Yin, Xiangyu Shen, Fucheng Li, Luyao Liu, Dao Sun

**Affiliations:** 1 Department of Breast and Thyroid Gland Surgery, Hunan Provincial Maternal and Child Health Care Hospital, Changsha, Hunan, China; 2 Department of Breast and Thyroid Gland Surgery, Hunan Provincial People’s Hospital, The First Affiliated Hospital of Hunan Normal University, Changsha, Hunan, China

**Keywords:** breast cancer, *LARP1*, macrophage polarization, prognostic biomarker, therapeutic target

## Abstract

**Background:**

Worldwide, breast cancer (BRCA) remains the most common cancer among women. Identifying novel biomarkers that not only predict prognosis but also inform therapeutic response is urgently needed. La-related protein 1 (*LARP1*), an RNA-binding protein and downstream effector of the PI3K/AKT/mTOR pathway, is related to multiple forms of cancer, though its impact on BRCA is not well understood.

**Methods:**

We performed a comprehensive investigation of *LARP1* in BRCA using multi-omics databases, including TCGA, GTEx and HPA. Functional annotations were conducted via STRING, GeneMANIA, and LinkedOmics platforms. The correlation between *LARP1* and tumor immunity was assessed through TISIDB and CIBERSORT. Experimental validation included RT-PCR, Western blotting, EdU, CCK-8, colony formation, transwell, ROS, JC-1, mPTP, and flow cytometry assays in *LARP1*-silenced SK-BR-3 and MDA-MB-231 cells, along with animal models and immunofluorescence on BRCA tissue microarrays.

**Results:**

*LARP1* was considerably overexpressed in BRCA tissues compared to normal counterparts and was associated with unfavorable overall and progression-free survival outcomes (OS and PFS). Functional enrichment indicated that *LARP1* participated in cell cycle progression, mitochondrial homeostasis, and immune modulation. Silencing of *LARP1* inhibited BRCA cells proliferation, migration, and invasion, and triggered mitochondrial dysfunction. Importantly, *LARP1* downregulation promoted the polarization of tumor-associated macrophages (TAMs) from the immunosuppressive M2 phenotype to the pro-inflammatory M1 phenotype. In clinical tissues, *LARP1* expression positively correlated with M2 macrophages and negatively with M1 macrophages.

**Conclusions:**

*LARP1* plays a pivotal oncogenic role in BRCA progression by enhancing cell proliferation and immune evasion through mitochondrial regulation and macrophage polarization. These findings highlight *LARP1* as a promising a potential therapeutic target, and diagnostic, immunological, prognostic biomarker for BRCA.

## Introduction

Among women, BRCA is the predominant form of cancer, with its rates climbing worldwide (1). In 2022, approximately 2.297 million new cases of BRCA were estimated, which made up 11.5% of all newly diagnosed cancers and about 23.8% of new instances of cancer in female patients (2). In the past few years, progress in molecular subtyping of BRCA, combined with the creation of targeted therapies and personalized treatment plans, has greatly enhanced the chances of survival and the standard of living for certain patients (3, 4). However, the heterogeneity of BRCA remains a major challenge in treatment, particularly among patients with metastatic disease, where significant differences in treatment response and frequent drug resistance result in limited overall therapeutic efficacy (5, 6). Research indicates that even with systemic treatment, the survival rate over five years for certain individuals with advanced or metastatic BRCA is under 20%, a figure much lower than that for those with early-stage BRCA (7). This indicates the restricted effectiveness of current therapeutic targets and biomarkers in some BRCA subtypes. Thus, there is a pressing necessity to identify more effective prognostic biomarkers to optimize personalized treatment strategies for BRCA.

*LARP1* belongs to a class of conserved RNA-binding proteins that are widely present in eukaryotic organisms (8, 9). thought to be a major player in post-transcriptional regulator that can bind to specific mRNA regions, such as the 3′ untranslated region (3′UTR) or 5′ terminal oligopyrimidine (5′TOP) sequences, thereby modulating the stability, localization, and translational efficiency of these mRNAs. It critically regulates the expression of various oncogenic transcripts (8). In recent years, *LARP1* has gradually attracted attention as a crucial downstream mediator in the PI3K/AKT/mTOR signaling pathway (10). This pathway serves as a critical axis for the regulation of cell proliferation, differentiation, apoptosis, and metabolism, and is highly activated in various malignant tumors (11). *LARP1* regulates intracellular protein synthesis rates and influences cell growth and division by selectively promoting or repressing the translation of 5′TOP mRNAs (such as ribosomal proteins and translation initiation factors like eIFs) through binding to these transcripts, a process modulated by mTORC1-mediated phosphorylation (12–14). Moreover, *LARP1* also plays an indispensable role in responding to cellular stress, maintaining mRNA stability, and regulating the adaptive survival of tumor cells (15, 16). Therefore, *LARP1* not only plays a vital role in fundamental RNA biology but may also exert central functions in cancer initiation, progression, and treatment resistance, making it a potential therapeutic target in oncology.

This research, which includes analyzes of public databases and experimental validation, demonstrated that There is a consistent overexpression of *LARP1* in BRCA tissues, which is significantly related to lower OS and PFS. These conclusions show that *LARP1* is important in the development of BRCA and acts as a potential indicator for prognosis, offering a theoretical foundation for clinical risk stratification and targeted therapy.

## Materials and methods

### Data download and survival analysis

Data on *LARP1* expression were gathered from various publicly available databases, including The Cancer Genome Atlas (TCGA, http://portal.gdc.cancer.gov/), the Genotype-Tissue Expression project (GTEx, https://www.gtexportal.org/), and the Gene Expression Omnibus (GEO, https://www.ncbi.nlm.nih.gov/geo/). Protein expression data for *LARP1* in human cancers were retrieved from the UALCAN portal (17) (http://ualcan.path.uab.edu/) and the Human Protein Atlas (18) (HPA, http://www.proteinatlas.org). The types of tumors included in the pan-cancer analysis were listed in **Supplementary Table 1**. Survival analysis was conducted using TCGA clinical data, and the “survival” package was used to analyze the connection between *LARP1* and patient results, such as OS and PFS.

### Assessment of immune infiltration

Cibersort, a method for deconvolution, estimates the composition of cell types in complex tissues using normalized gene expression profiles and has demonstrated strong alignment with ground truth estimations across various cancer types. We utilized Cibersort to measure the diversity of immune cell categories in different cancers and examined how *LARP1* correlates with these cells. Additionally, the TISIDB database (19) (http://cis.hku.hk/TISIDB/index.php) was utilized to systematically investigate the relationships between *LARP1* and various immune-related features in BRCA. To analyze the immune associations of *LARP1* in cancer, we examined and evaluated its correlation with the expression levels of immunoregulatory genes, including immunoinhibitors, immunostimulators, chemokines, and chemokine receptors.

### Genetic alteration analysis

Genetic alterations of the *LARP1* in BRCA were analyzed using the cBioPortal tool (20) (https://www.cbioportal.org/). The mutation sites of *LARP1* were visualized using the “Mutations” module, which provides both schematic diagrams and three-dimensional (3D) structural representations of protein alterations. Survival data were obtained and compared between BRCA patients with and without *LARP1* genetic alterations.

### Building nomograms for BRCA survival estimation

To anticipate the prognosis of BRCA patients, we built nomograms based on selected clinicopathological prognostic factors. The nomograms were created to predict the probabilities of 1/3/5 years OS. Calibration curves were constructed to examine the projected survival outcomes with the actual observed survival rates, thereby evaluating the accuracy of the nomogram model.

### STRINGS and GeneMANIA analysis

STRING (21) (www.string-db.org) serves as an internet database for the analysis of protein-protein interactions (PPI). We utilized the STRING platform to perform PPI network analysis of *LARP1* to investigate its functional associations in BRCA. GeneMANIA (22) (https://genemania.org/) was utilized to investigate gene function and construct a gene-gene interaction (GGI) network. The GGI network of *LARP1* was built based on the top 50 most significantly associated genes.

### Functional enrichment analysis

The LinkedOmics database (23) (http://www.linkedomics.org/login.php) was employed to analyze multi-omics data from TCGA database. The identification of differentially expressed genes connected to *LARP1* in BRCA was accomplished using the LinkFinder module. Pearson correlation analysis was performed, and the results were visualized through volcano plots and heatmaps. For functional annotation, the DEGs correlated with *LARP1* were subjected to Kyoto Encyclopedia of Genes and Genomes (KEGG) pathway and Gene Ontology (GO) enrichment analyzes.

### Drug sensitivity analysis

To evaluate the relationship between *LARP1* expression and sensitivity to anticancer therapies, we utilized the ROC plotter (24) (https://rocplot.com/) an open-access web resource that integrates transcriptomic data with treatment response information. This tool enables the assessment of biomarker efficacy in predicting drug responses. In this study, we analyzed datasets corresponding to both pathological complete response (PCR) and pathological treatment response in BRCA samples to explore the predictive value of *LARP1*.

### Cell culture and transfection

Human breast cancer cell lines, MDA-MB-231 and SK-BR-3, were cultured in RPMI-1640 medium (Gibco) supplemented with 10% fetal bovine serum (FBS) (Gibco) and 1% penicillin-streptomycin (Gibco) at 37 °C in a 5% CO_2_ incubator. For transfection, cells were plated at 70% confluency and transfected with siRNA (50 nM final concentration, Thermo Fisher Scientific) using Lipofectamine 2000 (Invitrogen), following the manufacturer’s instructions. The LARP1 siRNA (ID: S180095) was obtained from Thermo Fisher Scientific. Three siRNAs targeting LARP1 (siRNA1, siRNA2, siRNA3) were synthesized, and their sequences are listed in **Supplementary Table 2**. The GP-transfect-Mate reagent from GenePharma was used for transfection, following the manufacturer’s guidelines. The gene of *LARP1* was delivered using lentiviral transduction. Lentiviral particles were produced in HEK293T cells by co-transfecting the commercial transfer plasmid with the psPAX2 and pMD2.G packaging plasmids. Target cells were then transduced with the virus in the presence of polybrene, and stable cell pools were subsequently selected with puromycin. Successful transduction and expression were confirmed by qPCR and western blotting. Finally, we validated the knockout efficiency via qPCR and Western blot analysis.

### Protein extraction and Western blotting

Total protein was extracted using RIPA buffer (Thermo Fisher) supplemented with protease inhibitors (Roche, #04693132001) and phosphatase inhibitors (Thermo Fisher). Protein concentration was determined by the Bradford assay (Bio-Rad) using BSA as a standard. Equal amounts of protein (30 µg) were separated on 10% SDS-PAGE gels and transferred to PVDF membranes (Millipore). Blots were incubated with primary antibodies against LARP1 (Santa Cruz, #sc-376828), β-actin (Cell Signaling), and tubulin (Abcam), followed by HRP-conjugated secondary antibodies (Cell Signaling). Protein bands were detected using the ECL detection system (GE Healthcare) and quantified using ImageJ software.

### CCK-8 assay

To determine the seeding density of BRCA cells in a 96-well plate, cell counting was initially performed. Subsequently, 2,000 cells were seeded per well, with 3 to 6 replicate wells per group. A volume of CCK-8 (Dojindo) solution equivalent to 1/10 of the culture medium was added to each well. Concurrently, control wells containing the same volume of culture medium and CCK-8 reagent, and blank controls were set without cells. After incubation in a cell culture incubator for 2 h, the optical density at 450 nm was recorded. Following this, the absorbance was assessed at 24, 48, 72, and 96 h post-incubation with the microplate reader (BioTek, Synergy HTX).

### Colony formation assay

Cells in the logarithmic growth phase were enzymatically dissociated into single-cell suspensions. After counting, 500 cells were seeded per well in six-well plates and evenly distributed by gentle shaking. Cells were incubated at 37°C with 5% CO_2_ for 2–3 weeks, with medium replaced as needed. After colony formation, cells were stained with 0.1% crystal violet for 10 min following fixation in methanol for 15 min. The medium was first discarded, and after washing and air-drying, the colonies were photographed using a digital camera.

### Transwell migration and invasion assay

The Transwell chamber was pre-hydrated for 30 minutes at 37 °C with 50 μL of serum-free medium. Depending on whether an invasion or migration assay was being conducted, matrix gel was added to the upper chamber or left without. Cells in the logarithmic growth phase were collected, enzymatically dissociated, and counted, with 2,000 cells seeded into the upper chamber. The lower chamber was filled with 600 μL of medium containing 10% FBS. Gently swirl the culture plate to distribute cells evenly, then incubate at 37 °C in 5% CO_2_ for 24 hours. Following incubation, wash the chamber twice with calcium-free PBS, then fix with methanol for 30 minutes. Allow to air-dry, then stain with 0.1% crystal violet for 15 minutes. Gently wipe the upper surface to remove non-migrated cells, followed by three washes with PBS. Observe migrated cells under a microscope; randomly select three fields of view per chamber for counting, taking the average for statistical analysis.

### EdU staining

Cells in the logarithmic growth phase were plated into 6-well plates and incubated overnight. Cells were then incubated with a 2× EdU working solution (final concentration: 10 μM) for 2 h. After incubation, cells were fixed, washed, and permeabilized. Subsequently, 0.5 mL of click reaction mixture was added and allowed to react in the dark at room temperature for 30 min. After washing, nuclear staining was performed, and cells were observed under a fluorescence microscope.

### Detection of reactive oxygen species

Cells were treated with 10 μM 2′,7′-dichlorodihydrofluorescein diacetate (DCFH-DA; diluted 1:1000 in serum-free medium) at a concentration of 1-2 × 10^6^ cells/mL for 20 min at 37°C in a humidified incubator. During incubation, the cell suspension was gently turned over every 3–5 min to make sure adequate probe penetration and interaction. After the incubation period, remove any extra extracellular DCFH-DA. After si*LARP1* transfection and ROS probe loading, fluorescence intensity was monitored in real time using a fluorescence microscope.

### Flow cytometry

Flow cytometry on tissue samples requires the tissue to first be dissociated into single-cell suspensions. This is typically done by enzymatic digestion using enzymes such as collagenase or hyaluronidase, depending on the tissue type. After the tissue is minced into smaller pieces, it is incubated with the digestion enzyme (e.g., collagenase IV, 1 mg/mL) at 37 °C for 30–60 minutes to break down extracellular matrices and allow for cell dissociation. The resulting single-cell suspension is filtered through a 70 μm mesh to remove clumps and ensure single-cell flow cytometry analysis. Following cell dissociation, the cells are washed and stained with fluorochrome-conjugated antibodies specific to the cell markers of interest. Flow cytometry is then performed to analyze the expression of surface or intracellular markers in the cell populations. We use a BD FACSCanto II flow cytometer (BD Biosciences) to collect the data, and the results are analyzed using FlowJo software.

### Detection of mitochondrial membrane potential

After transfection with si*LARP1*, the cells were washed once with PBS and then incubated in 1 mL of culture medium supplemented with 1 mL of JC-1 working solution. The blend was carefully mixed, and the cells were then incubated at 37 °C in a humidified atmosphere for 20 min. Following incubation, the cells were washed twice with JC-1 working solution. Next, 2 mL of serum- and phenol red–containing medium was introduced to the cells. Fluorescence signals were observed using either a fluorescence microscope.

### Mitochondrial permeability transition pore assay

The cells were plated in 12-well plates and subjected to treatments as specified by the experimental protocols. Following PBS washing, the cells were subsequently treated with Calcein-AM staining solution and quenching solution at 37°C in darkness for 30 min. The staining solution was replaced with freshly pre-warmed medium, and cells were incubated again for 30 min to complete Calcein-AM hydrolysis. After PBS washes, fluorescence was observed under a fluorescence microscope after adding the detection buffer.

### M0 macrophage differentiation

THP-1 monocytes were cultured in RPMI-1640 medium with 10% FBS and 1% penicillin/streptomycin at 37°C in 5% CO_2_. For differentiation, cells were seeded at 5×10^5^ cells/mL and treated with 100 ng/mL PMA (Sigma-Aldrich) for 48 h. After incubation, the medium containing PMA was removed, cells were rinsed with PBS, and then cultured in new medium for 24 h to generate M0 macrophages.

### Multicolor immunoflow cytometry

M0 macrophages were co-cultured with shNC/shRNA MDA-MB-231 cells, and collected after 48 h, added pre-cooled Stain Buffer (FBS), centrifuged at 4 °C, 1200 rpm, for 5 min, discarded the supernatant; resuspend cells with 100 μL Stain Buffer (FBS), added Fc receptor blocking agent, incubated at 4 °C in the dark for 15 min, wash; resuspend cells with 50 μL Stain Buffer (FBS), added flow cytometry antibodies CD45 (eBioscience), CD11b (eBioscience), F4/80 (eBioscience), CD86 (eBioscience), CD206 (eBioscience) incubate at 4 °C in the dark for 0.5 h, wash; resuspend cells with Stain Buffer, and analyzed and visualized with Flowjo 10.8.1 software.

### Animal experiment

Ten six-week-old female BALB/c nude mice (China Jicui Biomedical) were maintained under standard conditions. Subcutaneous tumor models were established using shLARP1 MDA-MB-231 cells and shNC MDA-MB-231 cells (n=5 per group). A suspension of 2×10^6^ cells in PBS: Matrigel (1:1) was injected subcutaneously into the mice. Tumor volume was measured bi-weekly using calipers, and mice were euthanized upon reaching the study endpoint. At termination, tumor tissue was collected for multicolor immunofluorescence and immunohistochemistry (IHC) analysis to assess M1/M2 macrophage infiltration. Proliferation, apoptosis, and histopathological features were evaluated via hematoxylin and eosin (HE) staining, Ki67 staining, and TUNEL staining. Moreover, multicolor flow cytometry was performed on tumor tissues to assess the proportions of M1 and M2 macrophages. Mice were anaesthetized in a dedicated induction chamber using isoflurane (5% concentration, oxygen flow 1–1.2 L/min) until loss of consciousness was confirmed by the absence of the foot-withdrawal reflex. Euthanasia was then performed by continuous inhalation of isoflurane until respiratory arrest, with death confirmed by cervical dislocation.

### Verification in the tissue microarray

From a BRCA tissue microarray, a total of 70 paraffin-embedded samples of BRCA and the corresponding adjacent normal tissues were harvested. The process of multiplex immunofluorescence staining was executed with a four-color immunohistochemical staining kit (Absin, Catalog No. abs50013). Primary antibodies included *LARP1* (Proteintech), CD86 (Proteintech), and CD206 (Proteintech). Staining intensity was analyzed using a semiquantitative integration method with ImageJ software. Moreover, to calculate the correlation between the overall survival and *LARP1* expression.

### Statistics

Most of the numeric data are presented as mean ± SEM unless otherwise indicated. Statistical significance between two groups was determined by two-tailed Student’s t-test, while comparisons among multiple groups were performed using one-way ANOVA. Significance levels are indicated as follows: ***p < 0.001, **p < 0.01, and *p < 0.05. Statistical analyzes were conducted using GraphPad Prism v8.0.

## Result

### *LARP1* is highly expressed in BRCA

**Figure 1:**
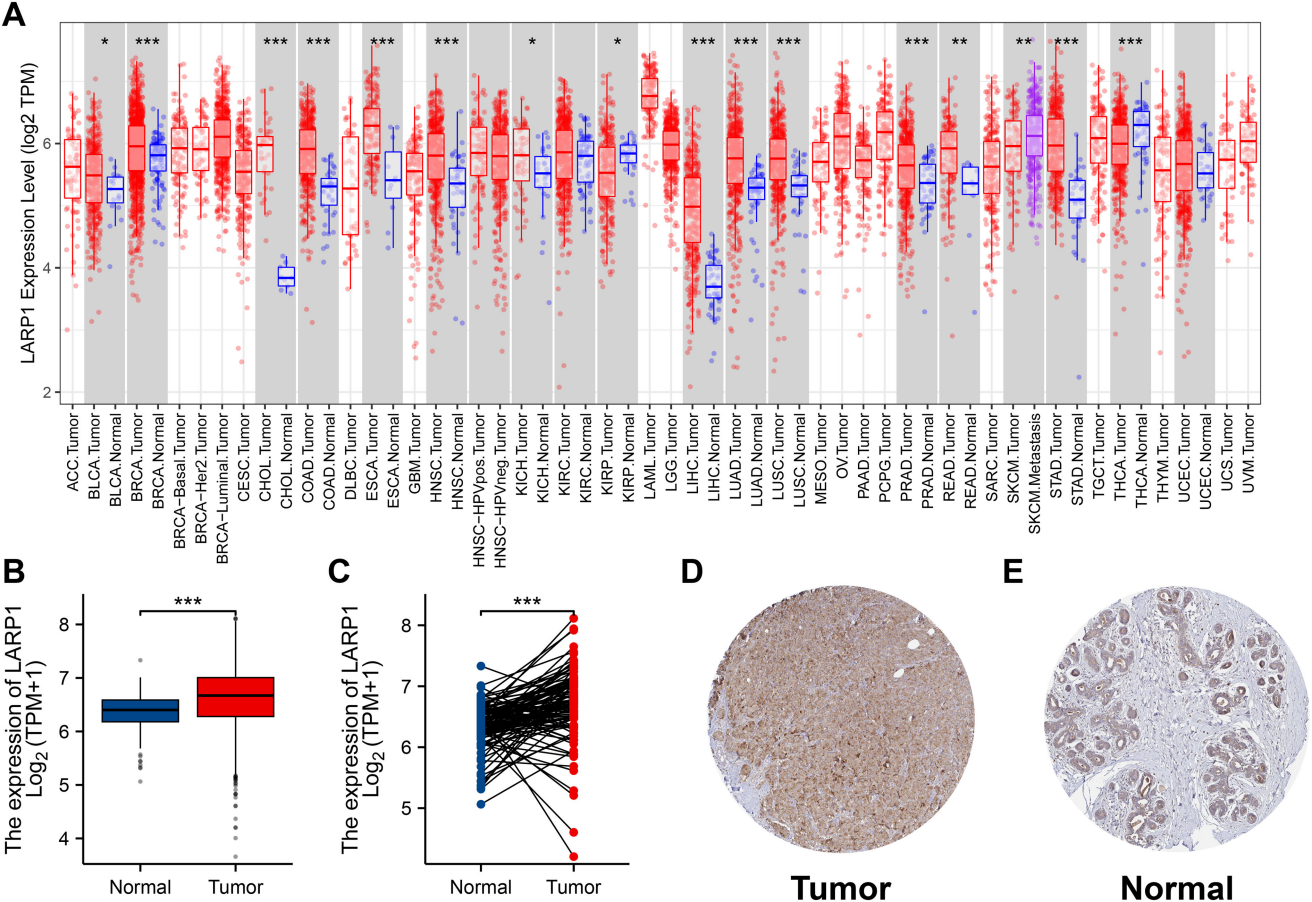
*LARP1* is highly expressed in breast cancer. **(A)** Pan-cancer overview of *LARP1* expression in tumor and normal tissues based on the TIMER2.0 database. **(B)** Comparison of *LARP1* expression between BRCA tissues and normal tissues based on integrated TCGA and GTEx data. **(C)** Paired comparison of *LARP1* expression between BRCA tissues and matched adjacent normal tissues. Representative immunohistochemical staining images of *LARP1* protein in BRCA tumor tissue **(D)** and normal breast tissue **(E)** from the HPA database. (*p< 0.05; **p< 0.01; ***p< 0.001).

To determine the expression pattern of *LARP1* in BRCA, we first performed a pan-cancer overview using the TIMER2.0 database. As shown in **Figure 1A**, *LARP1* was upregulated in several tumor types, including BRCA, compared with corresponding normal tissues. *LARP1* expression was modestly but significantly higher in BRCA than in normal breast tissue. Given the modest magnitude, we do not interpret fold-change alone as biological effect size; rather, we focus on the consistency across cohorts and the functional consequences of *LARP1* depletion. Focusing on BRCA, analysis integrating TCGA and GTEx datasets demonstrated that *LARP1* mRNA expression was significantly higher in BRCA tissues than in normal tissues (**Figure 1B**, P < 0.001). Paired analysis further confirmed that *LARP1* expression was markedly elevated in BRCA tissues compared with matched adjacent normal tissues (**Figure 1C**, P < 0.001). Consistently, immunohistochemical data from the HPA database showed stronger *LARP1* protein staining in BRCA tissues than in normal tissues (**Figures 1D, E**). In addition, the relationship between *LARP1* expression and clinicopathological characteristics in patients with BRCA is summarized in **Table 1**.

**Table 1:** Correlation between LARP1 expression and the clinicopathological features of BRCA cases.

Characteristics	Low expression of LARP1	High expression of LARP1	P value
n	543	544	
Pathologic T stage, n (%)			ns
T1	139 (12.8%)	139 (12.8%)	
T2	316 (29.2%)	315 (29.1%)	
T3	70 (6.5%)	70 (6.5%)	
T4	17 (1.6%)	18 (1.7%)	
Pathologic N stage, n (%)			ns
N0	270 (25.3%)	246 (23%)	
N1	167 (15.6%)	192 (18%)	
N2	53 (5%)	63 (5.9%)	
N3	45 (4.2%)	32 (3%)	
Pathologic M stage, n (%)			ns
M0	434 (46.9%)	471 (50.9%)	
M1	11 (1.2%)	9 (1%)	
Pathologic stage, n (%)			ns
Stage I	92 (8.7%)	90 (8.5%)	
Stage II	313 (29.4%)	306 (28.8%)	
Stage III	120 (11.3%)	124 (11.7%)	
Stage IV	11 (1%)	7 (0.7%)	
Race, n (%)			***
Asian	27 (2.7%)	33 (3.3%)	
Black or African American	122 (12.2%)	60 (6%)	
White	359 (36%)	396 (39.7%)	
Age, n (%)			ns
<= 60	294 (27%)	309 (28.4%)	
> 60	249 (22.9%)	235 (21.6%)	
Histological type, n (%)			**
Infiltrating Ductal Carcinoma	372 (37.9%)	404 (41.2%)	
Infiltrating Lobular Carcinoma	122 (12.4%)	83 (8.5%)	
ER status, n (%)			***
Negative	154 (14.9%)	86 (8.3%)	
Positive	368 (35.5%)	429 (41.4%)	
PR status, n (%)			***
Negative	207 (20%)	135 (13.1%)	
Positive	313 (30.3%)	379 (36.7%)	
HER2 status, n (%)			ns
Negative	274 (38.2%)	286 (39.9%)	
Positive	77 (10.7%)	80 (11.2%)	
PAM50, n (%)			***
LumA	282 (26.9%)	282 (26.9%)	
LumB	60 (5.7%)	146 (13.9%)	
Basal	122 (11.7%)	73 (7%)	
Her2	50 (4.8%)	32 (3.1%)	
Menopause status, n (%)			ns
Pre	101 (10.3%)	129 (13.2%)	
Peri	22 (2.3%)	18 (1.8%)	
Post	366 (37.5%)	340 (34.8%)	
Anatomic neoplasm subdivisions, n (%)			ns
Left	269 (24.7%)	297 (27.3%)	
Right	274 (25.2%)	247 (22.7%)	

ns > .05, *P < .05, **P < .01, ***P < .001.

### Prognostic significance of *LARP1* in BRCA and pan-cancer cohorts

**Figure 2:**
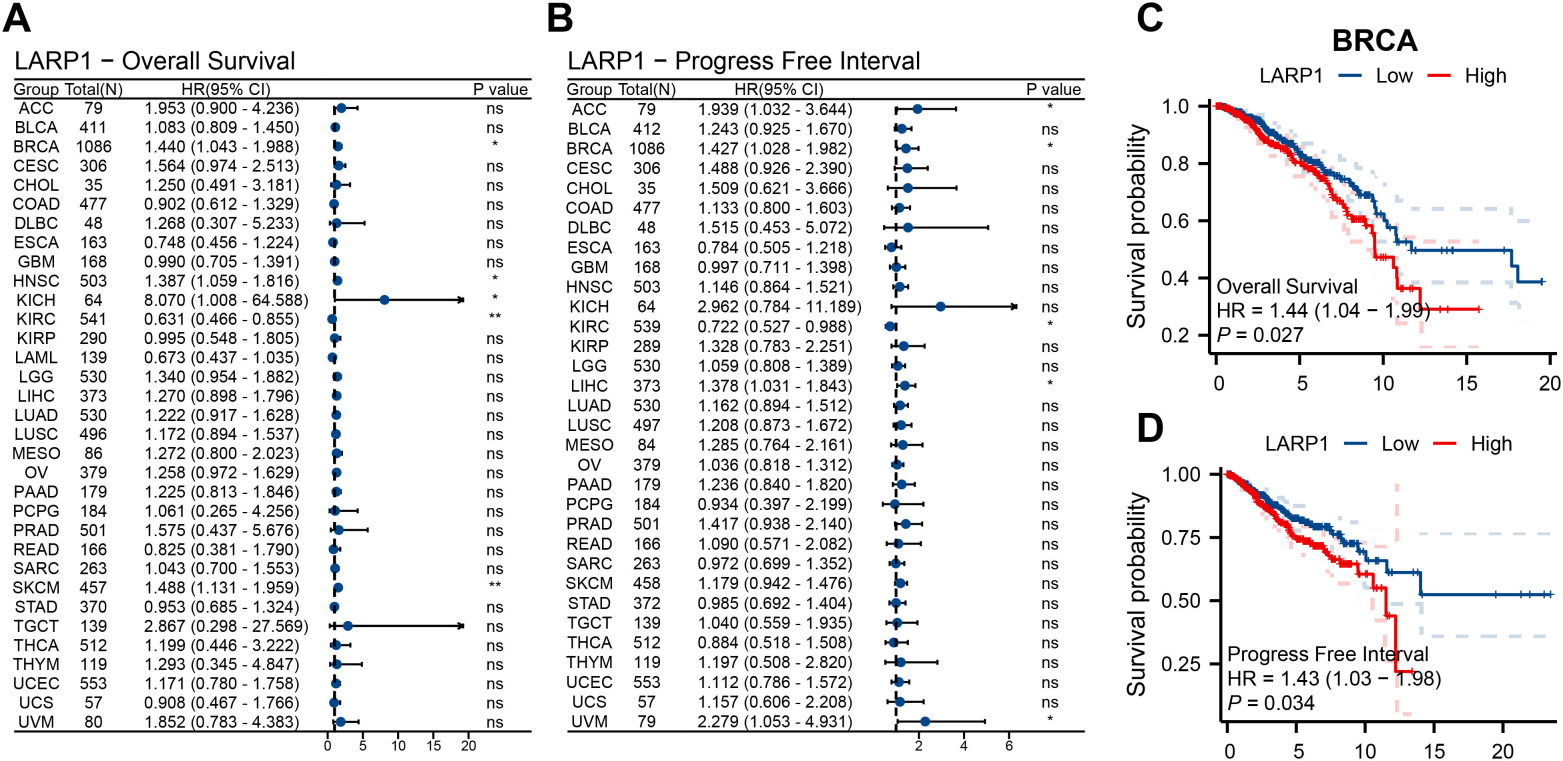
Prognostic significance of *LARP1* expression in pan-cancer cohorts and BRCA. **(A)** Forest plot summarizing the association between *LARP1* expression and OS across TCGA pan-cancer cohorts. **(B)** Forest plot summarizing the association between *LARP1* expression and PFI across TCGA pan-cancer cohorts. **(C)** Kaplan–Meier curve showing OS in BRCA stratified by high and low *LARP1* expression. **(D)** Kaplan–Meier curve showing PFI in BRCA stratified by high and low *LARP1* expression. (*p< 0.05; **p< 0.01).

We next investigated the prognostic value of *LARP1* across TCGA pan-cancer cohorts. As shown in **Figure 2A**, elevated *LARP1* expression was associated with poorer overall survival (OS) in several tumor types, including BRCA. Similarly, pan-cancer analysis of progress free interval (PFI) revealed that high *LARP1* expression was linked to shorter PFI in BRCA and several additional cancers (**Figure 2B**). Given our focus on breast cancer, we further assessed the survival impact of *LARP1* in BRCA. Kaplan–Meier analysis showed that patients with high *LARP1* expression had significantly reduced OS compared with those with low expression (**Figure 2C**, HR = 1.44, 95% CI: 1.04–1.99, P = 0.027). In agreement with this, increased *LARP1* expression also predicted shorter PFI in BRCA (**Figure 2D**, HR = 1.43, 95% CI: 1.03–1.98, P = 0.034). Collectively, these results indicate that *LARP1* is a potential adverse prognostic biomarker in BRCA. Subtype analysis further suggested that the prognostic impact of *LARP1* may vary across breast cancer molecular subtypes, with more evident associations observed in LumA and HER2-enriched tumors (**Supplementary Figure 1**).

### Genetic alteration of *LARP1*

**Figure 3:**
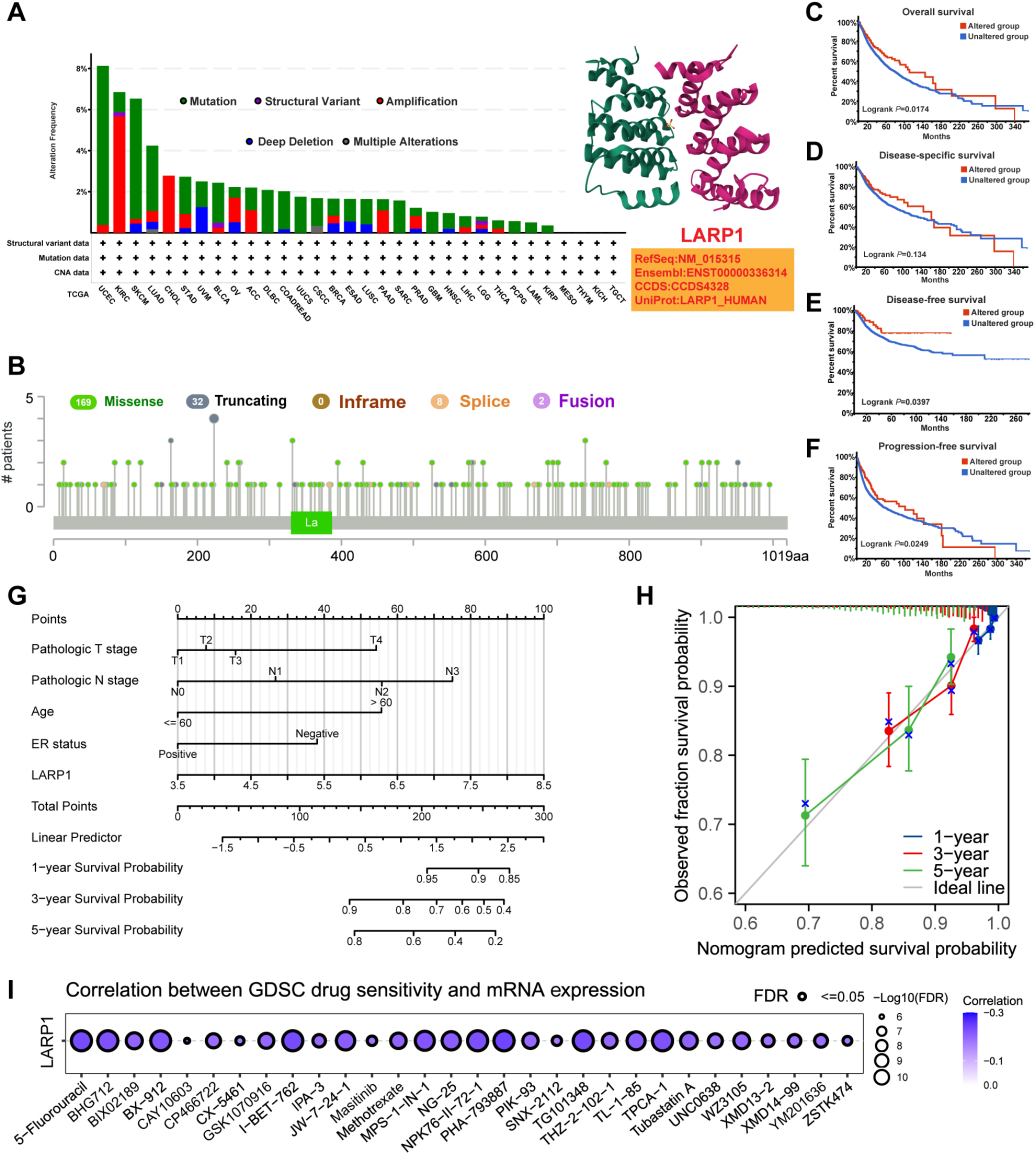
Genomic alterations, prognostic value, and drug resistance association of LARP1 in breast cancer. **(A)** Overview of *LARP1* genomic alterations across TCGA cancer types (pan-cancer), including mutation, structural variant, amplification, deep deletion, and multiple alterations. **(B)** Pan-cancer mutation landscape of LARP1 showing the distribution and classification of mutation types along the protein. **(C–F)** Kaplan-Meier survival analysis comparing OS **(C)**, DSS **(D)**, DFS **(E)**, and PFS **(F)** between *LARP1*-altered and unaltered groups in breast cancer patients. **(G)** Construction of a survival prediction nomogram for 1-, 3-, and 5-year timepoints, based on pathologic T/N stage, age, ER status, and *LARP1* expression. **(H)** Calibration plots showing consistency between nomogram-predicted and observed survival probabilities at 1, 3, and 5 years. **(I)** Correlation analysis between *LARP1* mRNA expression and drug sensitivity across a panel of chemotherapeutic and targeted agents in the GDSC database; darker circles indicate stronger correlation and significant false discovery rates (FDR <= 0.05).

The OncoPrint map in pan-cancer revealed that *LARP1* underwent various genetic alterations, including missense, truncating, inframe, splice, fusion mutations, and deep deletions (**Figure 3A**). In addition, the pan-cancer mutation landscape of LARP1 across multiple tumor types is summarized in **Figure 3B**, illustrating the distribution of alteration types and mutation sites along the protein. To assess the clinical significance of these alterations, we performed survival analysis in BRCA patients stratified by the presence or absence of *LARP1* alterations. The results demonstrated that BRCA patients harboring genetic alterations in *LARP1* exhibited significantly poorer DFS (P = 0.0397) and OS (P = 0.0174), whereas no major differences were detected in PFS (P = 0.0249) or DSS (P = 0.1340) when compared to patients without *LARP1* alterations (**Figures 3C–F**).

### Prognostic nomogram construction based on *LARP1* in BRCA

To evaluate the prognostic significance of *LARP1* in BRCA, we constructed a nomogram model using Cox proportional hazards regression via the RMS package in R (**Figure 3G**). The model incorporated five clinical variables: *LARP1* expression, age, estrogen receptor (ER) status, T stage, and N stage (**Table 2**). Each variable was assigned a weighted score, and the total score was used to estimate 1-, 3-, and 5-year OS probabilities in BRCA patients. The calibration analysis showed that the survival predictions from the nomogram were highly consistent with the actual observed results (**Figure 3H**), demonstrating the model’s good predictive accuracy.

**Table 2:** Univariate and multivariate Cox regression analysis of overall survival of patients with BRCA.

Characteristics	Total(N)	HR(95% CI) Univariate analysis	P value Univariate analysis	HR(95% CI) Multivariate analysis	P value Multivariate analysis
Pathologic T stage	1083				
T1	277	Reference		Reference	
T2	631	1.336 (0.890 - 2.006)	ns	1.208 (0.765 - 1.907)	ns
T3	140	1.551 (0.921 - 2.612)	ns	1.290 (0.716 - 2.324)	ns
T4	35	3.759 (1.959 - 7.213)	***	2.475 (1.208 - 5.071)	*
Pathologic N stage	1067				
N0	516	Reference		Reference	
N1	358	1.947 (1.322 - 2.865)	***	1.729 (1.138 - 2.629)	*
N2	116	2.522 (1.484 - 4.287)	***	2.644 (1.483 - 4.714)	***
N3	77	4.191 (2.318 - 7.580)	***	3.653 (1.916 - 6.965)	***
Age	1086				
<= 60	603	Reference		Reference	
> 60	483	2.024 (1.468 - 2.790)	***	2.572 (1.793 - 3.691)	***
ER status	1036				
Negative	240	Reference		Reference	
Positive	796	0.709 (0.493 - 1.019)	ns	0.534 (0.365 - 0.779)	**
LARP1	1086	1.387 (1.068 - 1.801)	ns	1.363 (1.032 - 1.799)	*

ns > .05, *P < .05, **P < .01, ***P < .001.

### Drug sensitivity analysis

The investigation into the sensitivity of various cancers to pharmacological agents, as mediated by *LARP1* expression levels, was conducted using the GSCA database. As showed in **Figure 3I**, an inverse association was observed between *LARP1* expression and 5-Fluorouracil, PHA-793887, BHG712 and so on. Negative correlation indicates a higher *LARP1* expression might make drug sensitive.

### ROC plotter analysis for chemotherapy and targeted therapy

To further evaluate the predictive potential of *LARP1* expression for treatment efficacy in BRCA, we analyzed its association with pathological complete response (PCR) across various molecular subtypes and therapeutic regimens using ROC curve analysis. Specifically, *LARP1* showed notable predictive power in HER2+ subtypes, with the highest AUC observed in the HER2+ Lapatinib group (AUC = 0.792, **Supplementary Figure 2M**), followed by HER2+ FEC (AUC = 0.719, **Supplementary Figure 2I**), HER2+ ER− FEC (AUC = 0.677, **Supplementary Figure 2H**), and HER2+ Anthracycline (AUC = 0.641, **Supplementary Figure 2K**). In luminal subtypes, *LARP1* demonstrated moderate discriminative ability for predicting response to FEC (AUC = 0.708, **Supplementary Figure 2R**), Anthracycline (AUC = 0.697, **Supplementary Figure 1O**), and Taxane (AUC = 0.663, **Supplementary Figure 2Q**) in luminal A or B patients. In ER+ BRCA cohorts, *LARP1* predicted therapeutic response to FEC (AUC = 0.687, **Supplementary Figure 2B**), Anthracycline (AUC = 0.618, **Supplementary Figure 2A**), and Trastuzumab (AUC = 0.605, **Supplementary Figure 2D**) with modest performance. Notably, triple-negative BRCA patients showed relatively poor AUCs (0.592-0.560, **Supplementary Figure 2S, 2T**), suggesting limited predictive value of *LARP1* in this subgroup. Overall, these results indicate that *LARP1* may serve as a potential biomarker for predicting treatment response in specific BRCA subtypes, particularly in HER2+ and luminal-type patients receiving targeted therapies or anthracycline-based regimens.

### Expression network and functional enrichment analysis

**Figure 4:**
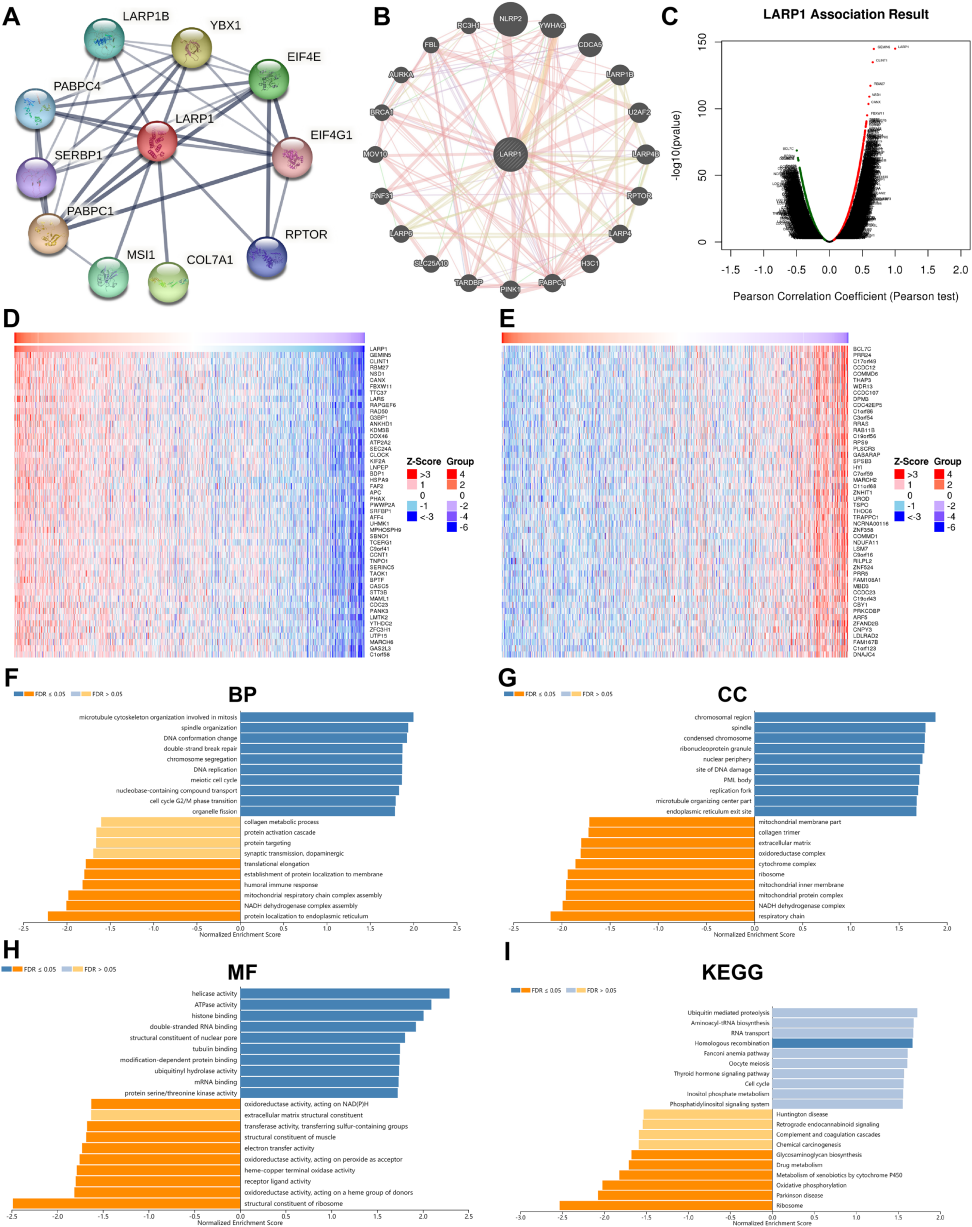
Functional network, gene co-expression, and enrichment analysis of *LARP1* in breast cancer. **(A)** PPI network analysis of *LARP1* performed through the STRING database, showing its association with key RNA-binding and translational regulatory protein. **(B)** Gene-gene interaction network centered on *LARP1* using GeneMANIA, indicating biological co-expression and physical interaction relationships. **(C)** Volcano plot of *LARP1* co-expressed genes based on Pearson correlation coefficients and P-values. **(D, E)** Heatmaps depicting the 50 strongest negative **(D)** and positive **(E)** correlations with *LARP1* expression in BRCA samples. **(F–H)** GO enrichment of *LARP1*-related genes in BP, CC and MF. **(I)** KEGG pathway analysis indicated that genes associated with *LARP1* participate in oncogenic pathways, including cell cycle, RNA degradation, mTOR signaling, ubiquitin-mediated proteolysis, and p53 signaling pathway.

We conducted a PPI network analysis using STRING, which revealed that *LARP1* interacts with several RNA-binding and translation-related proteins (e.g., LARP1B, YBX1, EIF4E) (**Figure 4A**). Co-expression network analysis based on TCGA-BRCA data showed that *LARP1* is positively associated with cell cycle– and mitosis-related genes (e.g., CDC45, MCM5, PLK1), as shown in the interaction network and volcano plot (**Figures 4B, C**). Heatmap analysis indicated distinct expression patterns between high- and low-*LARP1* groups (**Figures 4D, E**). To further explore functional implications, GO and KEGG enrichment analyzes were performed. In the BP category, *LARP1-*associated genes showed significant enrichment in chromosome organization, DNA replication, and cell cycle phase transition (**Figure 4F**). In the CC category, they were associated with condensed chromosomes, kinetochore, and mitotic spindle (**Figure 4G**). In the MF category, a notable enrichment was detected in helicase activity, ATPase activity, and RNA binding (**Figure 4H**). KEGG pathway analysis further highlighted cell cycle, RNA transport, p53 signaling, mTOR signaling, and ubiquitin-mediated proteolysis as major enriched pathways (**Figure 4I**).

### Relationship between *LARP1* and immunity

**Figure 5:**
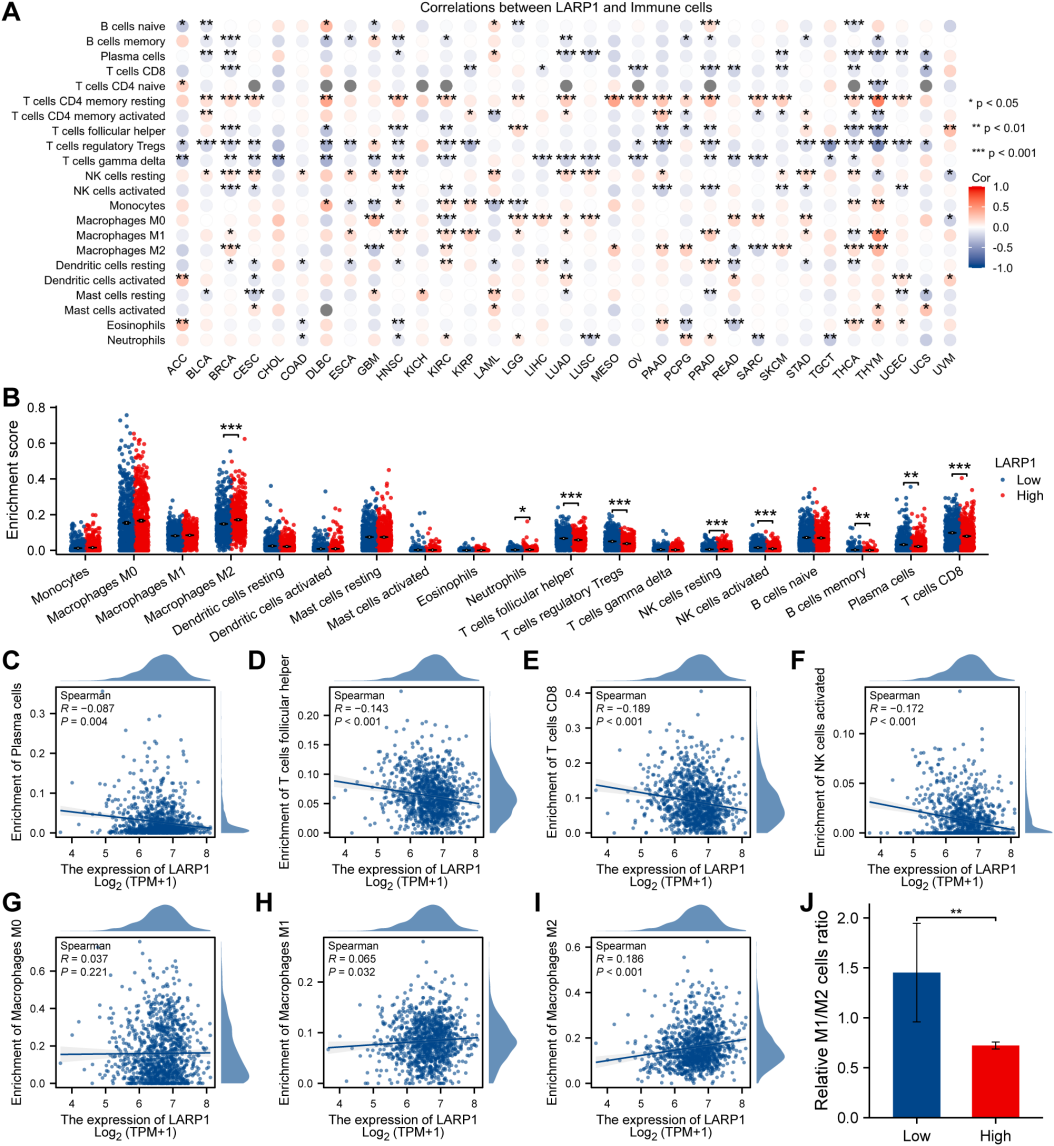
Correlation between *LARP1* and immune cell infiltration in BRCA. **(A)** Heatmap showing the Spearman correlation coefficients between *LARP1* expression and 22 immune cell types across various TCGA cancer types. **(B)** Immune cell enrichment score analysis between BRCA samples with high and low *LARP1* expression. **(C–I)** Spearman correlation scatter plots showing negative correlations between *LARP1* expression and plasma cells **(C)**, T follicular helper cells **(D)**, CD8+ T cells **(E)**, activated NK cells **(F)**, and positive correlation with M2 macrophages **(I)**. **(J)** Quantification of M1/M2 macrophage ratio in high vs. low LARP1 expression groups. (*p< 0.05; **p< 0.01; ***p< 0.001).

**Figure 6:**
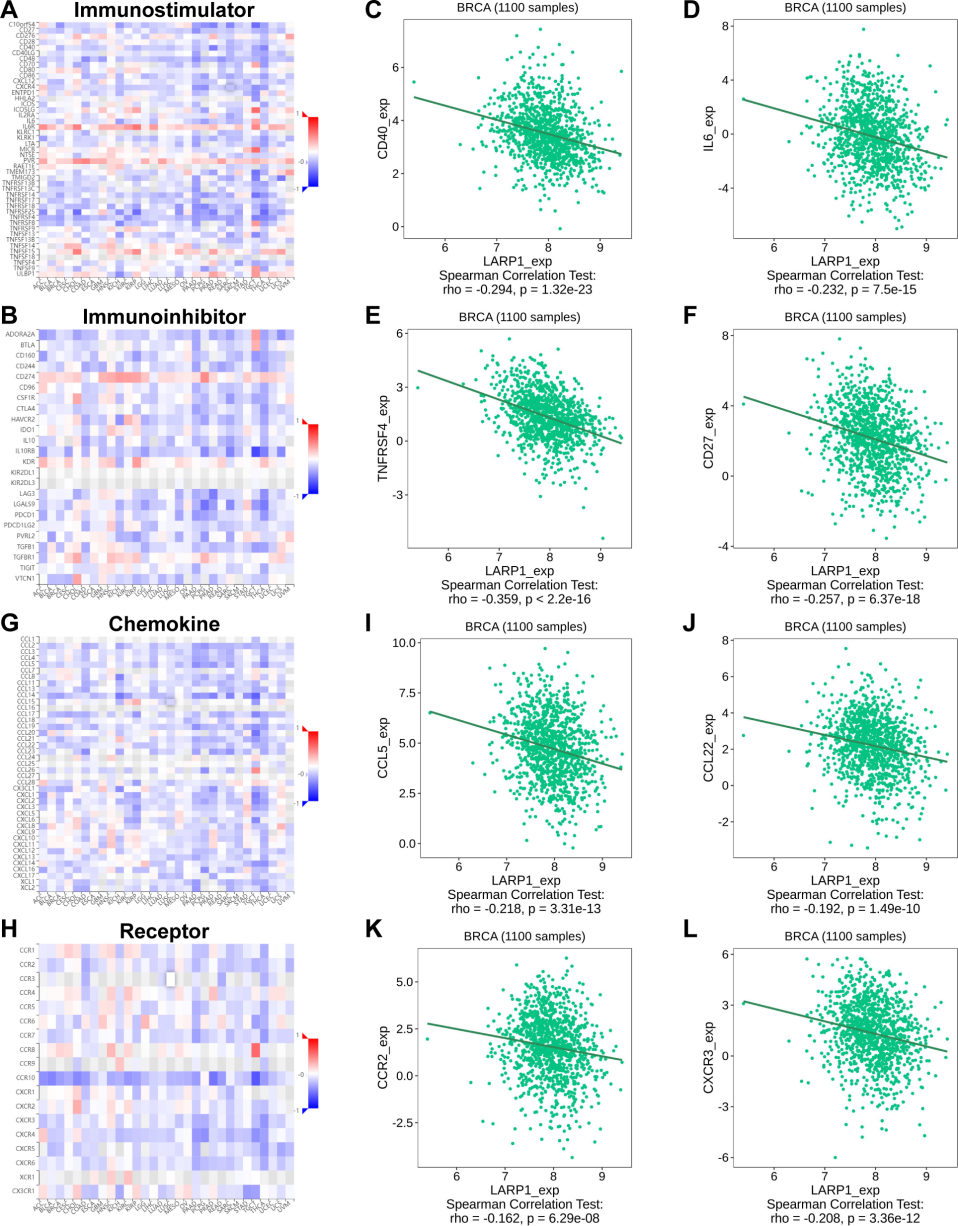
Correlation between *LARP1* expression and immune regulatory molecules in breast cancer. **(A, B)** Correlation heatmaps of *LARP1* expression with immune stimulators **(A)** and immune inhibitors **(B)** across 1,100 BRCA samples from TCGA. **(C–F)** Scatter plots illustrating significant negative correlations between *LARP1* and immune stimulatory molecules CD40 **(C)**, IL6 **(D)**, TNFRSF4 **(E)**, and CD27 **(F)** (all P < 0.001). **(G, H)** Heatmaps showing correlations between *LARP1* and chemokines **(G)** and chemokine receptors **(H)** in BRCA samples. **(I–L)** *LARP1* expression is inversely correlated with several chemokines and their receptors, including CCL5 **(I)**, CCL22 **(J)**, CCR2 **(K)**, and CXCR3 **(L)**.

To investigate the immune relevance of *LARP1* in BRCA, the association between *LARP1* expression and immune cell infiltration was evaluated using heatmaps and the TISIDB database (**Figure 5A**). In BRCA patients, reduced *LARP1* expression was linked to significantly higher infiltration of CD8^+^ T cells, plasma cells, NK cells (activated), follicular helper T cells, and memory B cells, along with a higher M1/M2 macrophage ratio (**Figures 5B–J**). Conversely, high *LARP1* expression correlated with increased M2 macrophages and resting NK cells. We also observed significant negative correlations between *LARP1* expression and immune stimulatory/inhibitory molecules such as CD40, IL-6, TNFRSF4, and CD27 (**Figures 6A, B**). Further analysis demonstrated a negative correlation between LARP1 expression and chemokine levels (e.g., CCL5, CCL22) and receptors (e.g., CCR2, CXCR3) in BRCA (**Figures 6C–L**). These findings indicate that *LARP1* may promote an immunosuppressive tumor microenvironment by modulating immune cell infiltration and migration. Immune infiltration varied markedly across Luminal, HER2-enriched, Basal-like tumors and normal breast tissues, with most immune subsets showing significant inter-group differences (**Supplementary Figure 3**). Basal-like tumors were relatively enriched for macrophage M0, HER2-enriched tumors for activated NK cells, and luminal tumors for Tregs/activated mast cells, whereas CD4^+^ naïve T cells and eosinophils showed no significant differences.

### *LARP1* knockdown suppresses tumor cell growth and motility

**Figure 7:**
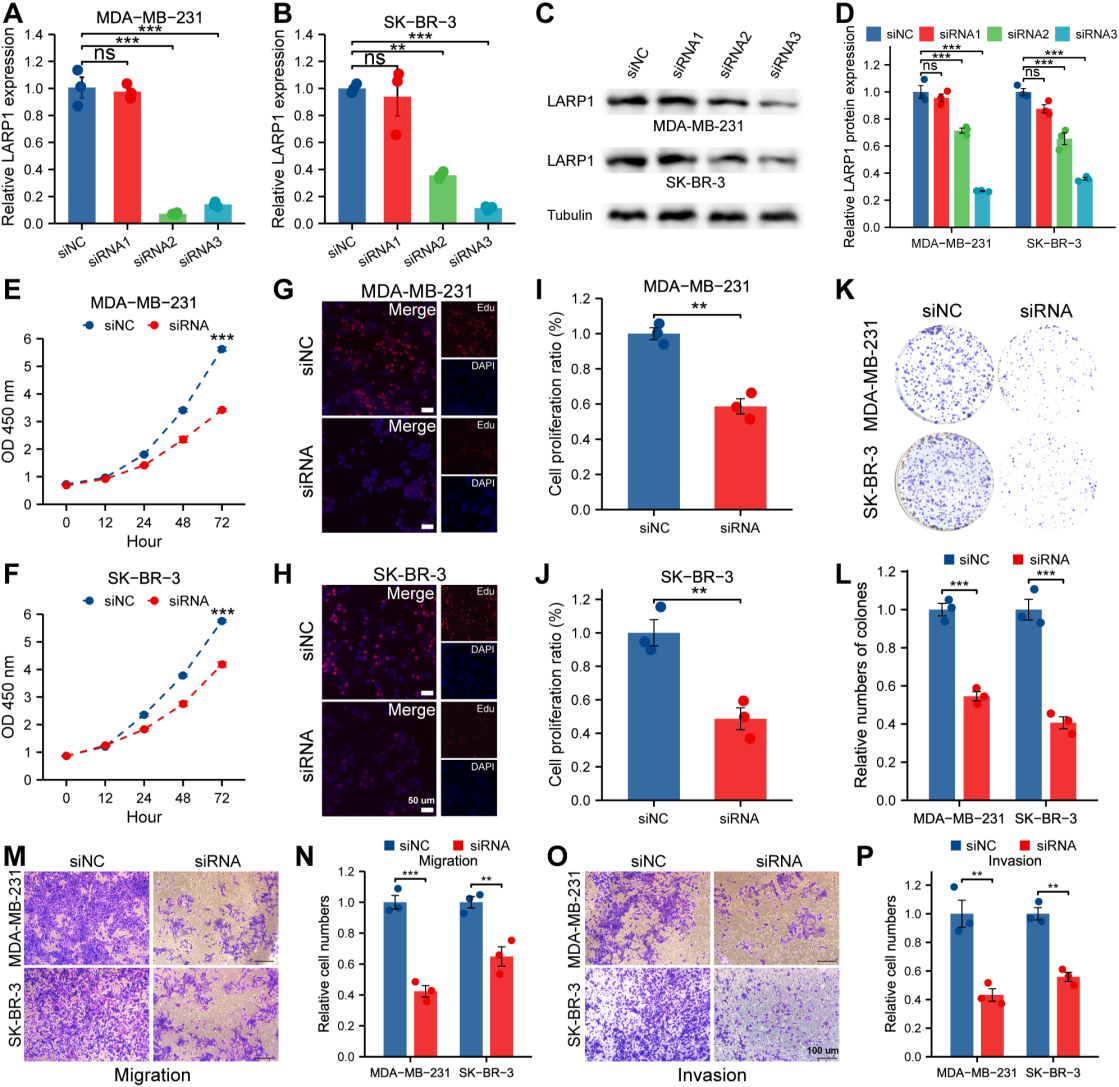
Silencing *LARP1* suppresses the proliferative, migratory, and invasive capabilities of breast cancer cells. **(A, B)** Quantitative RT-PCR analysis confirmed efficient knockdown of *LARP1* mRNA by siRNA1, siRNA2, and siRNA3 in MDA-MB-231 **(A)** and SK-BR-3 **(B)** cells. **(C, D)** Western blot and densitometric analysis showing reduced *LARP1* protein expression upon siRNA transfection in both cell lines. **(E, F)** CCK-8 cell viability assay demonstrated that *LARP1* knockdown significantly inhibited proliferation of MDA-MB-231 **(E)** and SK-BR-3 **(F)** cells. **(G–J)** EdU assay images **(G, H)** and quantification **(I, J)** further confirmed that *LARP1* silencing inhibited DNA replication and cell proliferation. **(K-L)** Colony formation assays and statistical analysis showing that *LARP1* knockdown significantly impaired the clonogenic ability of MDA-MB-231 and SK-BR-3 cells. **(M, N)** Representative images **(M)** and quantification **(N)** of transwell migration assays indicated reduced migratory capacity following *LARP1* depletion. **(O, P)** Transwell invasion assay images **(O)** and corresponding quantification **(P)** revealed decreased invasive potential of cells transfected with *LARP1* siRNA compared to negative control (siNC). (**p< 0.01; ***p< 0.001).

To investigate the biological functions of *LARP1* in BRCA, siRNA-mediated knockdown models were successfully established in SK-BR-3 and MDA-MB-231 cells, as confirmed by RT-PCR and Western blotting (**Figures 7A–D**). The CCK-8 and EdU proliferation tests showed that reducing *LARP1* expression greatly decreased the proliferation ability of both cell lines (**Figures 7E–J**). Furthermore, colony formation assays showed that *LARP1* knockdown markedly reduced the clonogenic potential of BRCA cells (**Figures 7K, L**). In addition, Transwell assays demonstrated that *LARP1* silencing significantly impaired both the invasive and migratory capabilities of BRCA cells (**Figures 7M–P**).

### Silencing *LARP1* promotes ROS accumulation and mPTP opening

**Figure 8:**
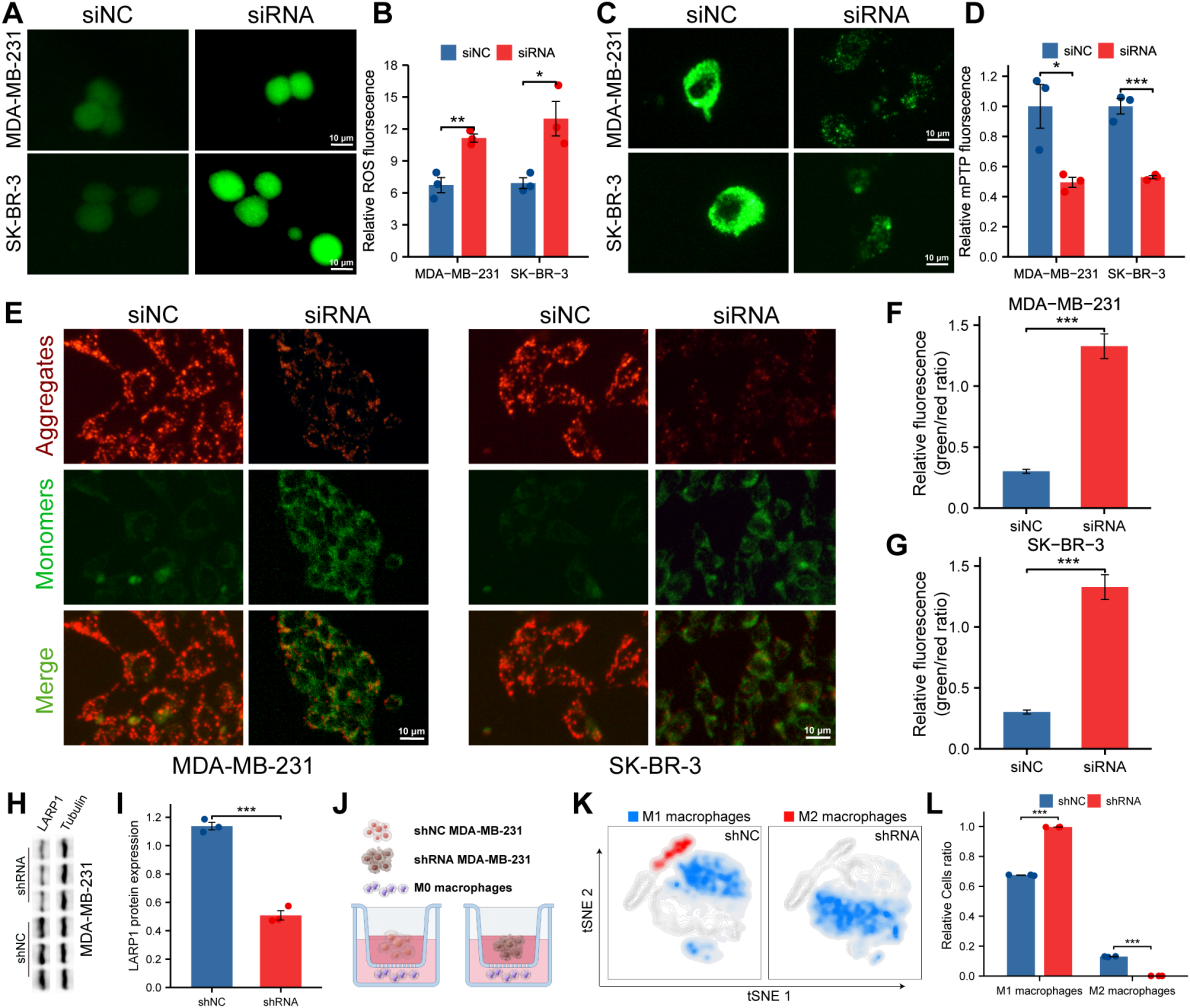
*LARP1* knockdown induces mitochondrial dysfunction and promotes macrophage M1 polarization. **(A, B)** Representative fluorescence images **(A)** and quantification **(B)** showing increased intracellular ROS levels following *LARP1* knockdown. **(C, D)** Fluorescence microscopy **(C)** and quantification **(D)** revealed reduced mitochondrial permeability transition pore (mPTP) opening after *LARP1* knockdown. **(E–G)** JC-1 staining images **(E)** and the green/red fluorescence ratio **(F, G)** showed a marked decrease in mitochondrial membrane potential (ΔΨm) in both cell lines upon *LARP1* silencing. **(H–I)** Western blot and densitometric analysis confirmed efficient knockdown of *LARP1* protein in MDA-MB-231 cells using shRNA. **(J)** Schematic of macrophage polarization assay based on co-culture of MDA-MB-231 cells with monocytes. **(K–L)** tSNE plots **(K)** and statistical analysis **(L)** of flow cytometry results showed that *LARP1* depletion enhanced M1 macrophage polarization (CD86^+^) and suppressed M2 polarization (CD206^+^). (*p< 0.05; **p< 0.01; ***p< 0.001).

Upon knockdown of *LARP1*, we employed ROS assay kit to assess ROS levels in BRCA cells, revealing a marked elevation in ROS content (**Figures 8A, B**). Moreover, we assessed the mPTP in BRCA cells and found that knocking down *LARP1* significantly induced mPTP opening (**Figures 8C, D**). Additionally, utilizing the JC-1 assay kit, a significant decrease in mmp was observed in BRCA cells following *LARP1* knockdown (**Figures 8E–G**). These findings validate that targeting BRCA *LARP1* may induce mitochondrial dysfunction.

### *LARP1* knockdown enhances M1 macrophage polarization

We have successfully constructed a stable *LARP1* knockout MDA-MB-231 cells (**Figures 8H, I**). To validate the impact of *LARP1* knockdown in sh*LARP1* MDA-MB-231 cells on macrophage polarization, we constructed a co-culture system (**Figure 8J**). Flow cytometry analysis revealed that *LARP1* low-expression BRCA cells were associated with a significantly increased M1/M2 macrophage ratio (**Figures 8K, L**). This finding suggested that targeting *LARP1* may provide a therapeutic strategy for regulating immune response.

### *LARP1* knockdown reduces tumor burden and promotes immune activation *in vivo*

**Figure 9:**
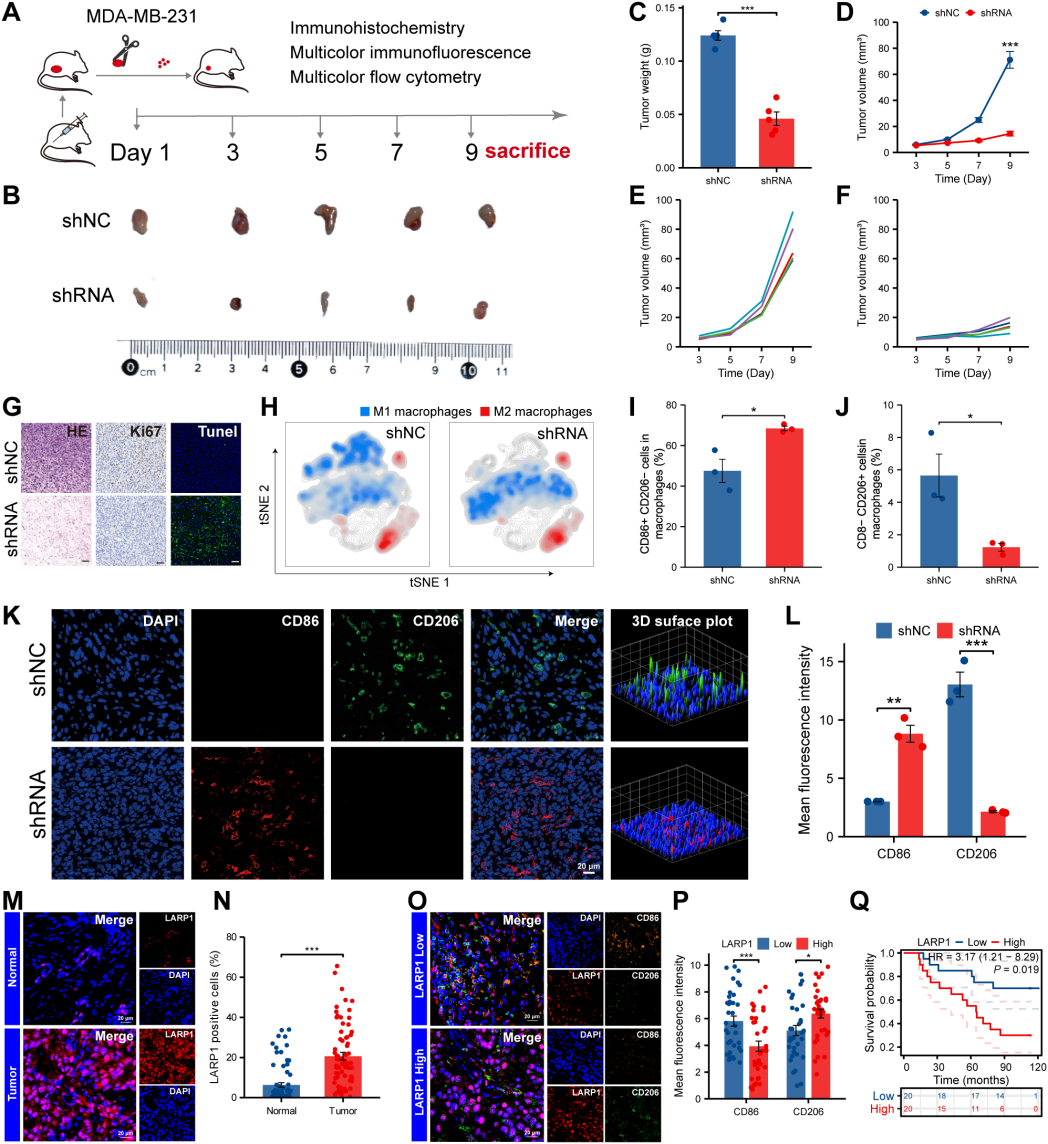
Silencing of *LARP1* suppresses tumor progression and alters macrophage polarization *in vivo*. **(A)** Experimental design schematic. **(B)** Tumor images from shNC and shLARP1 MDA-MB-231 groups (n=5). **(C, D)** Tumors in the sh*LARP1* group showed significantly lower weights than controls and tumor volume curves indicating growth inhibition by *LARP1* knockdown. **(E–F)** Individual growth curves for each mouse. **(G)** H&E, Ki67, and TUNEL staining showed decreased proliferation and increased apoptosis in sh*LARP1* tumors. **(H–J)** t-SNE plots of M1 and M2 macrophage distribution and flow cytometry quantification of CD86^+^CD206^−^ (M1) and CD206^+^CD86^−^ (M2) macrophages. **(K–L)** Immunofluorescence staining of CD86 and CD206 in tumor sections and quantification of CD86 and CD206 fluorescence intensity. **(M, N)** Immunofluorescence staining of *LARP1* in normal and tumor tissues and quantification of *LARP1*^+^ cells in both tissues (n=70). **(O, P)** Multiplex immunofluorescence showing CD86, CD206, and *LARP1* in tumors with low vs. high *LARP1* expression and quantitative comparison of CD86 and CD206 in high vs. low *LARP1* groups (n=70). **(Q)** Kaplan-Meier survival curves showing that high *LARP1* expression correlates with poor OS in patients with BRCA (n=40). (*p< 0.05; **p< 0.01; ***p< 0.001).

A subcutaneous tumorigenesis experiment was conducted in mice using the shNC/sh*LARP1* MDA-MB-231 cell line (**Figure 9A**). The results showed that cell growth *in vivo* slowed down after *LARP1* knockdown. This conclusion was further confirmed by the significant reduction in tumor size and weight *in vitro* (**Figures 9B–F**). Moreover, H&E staining, Ki67 immunohistochemistry, and TUNEL staining revealed that *LARP1* knockdown led to decreased tumor cell proliferation and increased apoptosis in tumor tissues (**Figure 9G**). In addition, we proved by flow cytometry (**Figures 9H–J**) and multiplex immunohistochemistry (**Figures 9K, L**) analysis that knockout genes can induce M2 macrophages to polarize to M1 macrophages.

### *LARP1* expression and immunological responses in the tissue microarray

Multiplex immunofluorescence analysis revealed significantly elevated *LARP1* expression in BRCA tissues compared to adjacent normal tissues (**Figures 9M, N**, P < 0.001). Stratified analysis of tumor sections based on *LARP1* expression levels demonstrated that high *LARP1* expression was associated with a decrease in CD86^+^ M1 macrophages and an increase in CD206^+^ M2 macrophages infiltration (**Figures 9O, P**, P < 0.001 and P < 0.05, respectively). Patients with increased *LARP1* expression showed a substantially worse OS than those with lower expression, as determined by Kaplan–Meier survival analysis (HR = 3.17, P = 0.019; **Figure 9Q**).

## Discussion

*LARP1*, a member of the LARP family, is able to bind RNA and influence gene expression following transcription (9, 25). Recent research indicates that *LARP1* is crucial in the development and advancement of cancer, primarily by influencing mRNA stability, translational regulation, and cellular signaling pathways (10, 26). Nonetheless, its importance in biology and clinical implications in BRCA are still not well understood. In this study, we systematically investigated the expression patterns, prognostic significance, functional networks, and immune-related characteristics of *LARP1* in BRCA through integrated bioinformatics analysis and experimental validation.

BRCA tissues exhibited a significant upregulation of *LARP1* expression when compared to adjacent normal tissues. Further evidence for this trend was provided by immunohistochemistry data from the HPA database. These results align with research conducted on hepatocellular carcinoma, ovarian cancer, and lung cancer. In hepatocellular carcinoma, poor prognosis is connected to high *LARP1* expression (27). In ovarian cancer, *LARP1* maintains mitochondrial energy metabolism by regulating the mRNA stability and translational efficiency of mitochondrial oxidative phosphorylation-related proteins, such as TFB2M and SDHB, thereby promoting tumor cell survival and reducing sensitivity to PI3K/mTOR inhibitors (26). In non-small cell lung cancer, *LARP1* facilitates epithelial-mesenchymal transition, which increases the invasiveness and ability of tumor cells to metastasize (28).

We also analyzed the genetic alterations of *LARP1*. The mutation frequency of *LARP1* was approximately 2.5%, including missense mutations, truncating mutations, and gene amplifications. BRCA patients with *LARP1* mutations showed significantly poorer OS, DFS, and PFS than those without. The prognostic nomogram model and its calibration curves showed a high consistency between the forecasted survival chances for 1, 3, and 5 years and the real results, indicating that *LARP1* expression can serve as a valuable indicator for individualized prognosis prediction in BRCA. In addition, the GDSC database analysis of drug sensitivity indicated a negative correlation with LARP1 expression. and sensitivity to various chemotherapeutic and targeted drugs, indicating that *LARP1* might influence drug resistance in BRCA.

To explore the potential mechanisms underlying its tumor-promoting effects, our study included analyzes of gene co-expression and functional enrichment. GO enrichment revealed that *LARP1*-associated genes were highly enriched in BP such as DNA replication, cell cycle regulation, mitosis, and chromosome segregation. In terms of CC, these genes were enriched in chromosomal regions, condensed chromosomes, and kinetochores. Regarding MF, the enriched functions included helicase activity, DNA binding activity, and ATPase activity. These findings suggested that *LARP1* is closely associated with cell cycle progression and genomic stability in tumor cells, highlighting its critical role in cancer development. KEGG pathway enrichment analysis indicated that LARP1-related genes participate in multiple classical oncogenic signaling pathways—such as the cell cycle, p53 signaling, RNA degradation, mTOR signaling, and ubiquitin-mediated proteolysis—aligning with the observations reported by Tcherkezian et al. and Mura et al. (14, 26).

CCL5 can drive macrophages to polarize into the M1 phenotype and boost T cell infiltration along with the IFN-γ response (29, 30). CCL5 promotes the expression of M1-associated genes such as iNOS, IL-12, and TNF-α by binding to its receptors (e.g., CCR1, CCR3, and CCR5) and activating signaling pathways including NF-κB and STAT1 (29). Our analysis demonstrated that *LARP1* expression was significantly inversely correlated with CCL5 levels. Notably, in BRCA tissues with high *LARP1* expression, CCL5 levels were reduced, suggesting that *LARP1* may suppress the CCL5 signaling axis, thereby impairing M1 macrophage polarization and promoting tumor immune evasion. Previous studies have indicated that high expression of the glucose-metabolizing enzyme *GPI* is also closely associated with dysregulated immune infiltration and poor prognosis in BRCA (31). The study revealed that increased expression of *LARP1* in BRCA facilitates the creation of an immunosuppressive environment within the tumor, specifically manifested by enhanced the polarization of M2 macrophages and downregulation of immune-stimulatory molecules such as CD40. This phenomenon is highly consistent with recent findings on *RPGRIP1L* (32). CD40, as a key molecule involved in immune co-stimulation, is extensively found on antigen-presenting cells and is crucial in its function in regulating the polarization from M2 to M1 macrophages (33). Previous studies have demonstrated that CD40 activation markedly increases the production of pro-inflammatory cytokines (e.g., IL-12) and enhances antigen-presenting capacity via CD40L-mediated signaling pathways, such as NF-κB, JAK/STAT, and MAPK. This process promotes the polarization of macrophages from the M2 macrophages toward the M1 macrophages (34, 35).

Recent studies have demonstrated that LARP1 directly regulates the stability and translational efficiency of mRNAs encoding key mitochondrial oxidative phosphorylation (OXPHOS) components, thereby sustaining mitochondrial respiration and ATP production under oncogenic stress conditions (36); accordingly, in ovarian cancer models, LARP1 depletion impairs OXPHOS activity, induces mitochondrial membrane depolarization, triggers excessive ROS accumulation, and sensitizes tumor cells to metabolic stress and apoptosis (26). Consistent with these findings, our data show that silencing LARP1 in breast cancer cells markedly induces mitochondrial dysfunction—manifested by elevated intracellular ROS, loss of mitochondrial membrane potential (ΔΨm), and enhanced mPTP opening—hallmarks of bioenergetic collapse and redox imbalance that support an essential role for LARP1 in maintaining mitochondrial integrity and restraining oxidative-stress vulnerability in BRCA cells. Importantly, LARP1 downregulation also reshapes the tumor–immune interface: because macrophage phenotypes were assessed using direct co-culture followed by flow cytometry, the polarization changes likely reflect an integrated outcome of tumor–macrophage contact and tumor-derived soluble signaling rather than a single isolated mechanism; in line with prior evidence that disruption of oncogenic PI3K/mTOR signaling can promote M1 markers and suppress tumor progression (37). we observed that LARP1 downregulation in MDA-MB-231 cells increased M1-like macrophages (CD86^+^; a pro-inflammatory marker (38)) while reducing M2-like macrophages (CD206^+^; associated with IL-10 secretion and immunosuppression (39, 40). Moreover, tissue microarray multiplex immunofluorescence further confirmed that LARP1 is upregulated in BRCA tissues and positively correlates with M2 infiltration but negatively correlates with M1 macrophages, and given that FOXP3 (a key determinant of Treg function) is closely associated with BRCA prognosis (41) these findings collectively highlight that LARP1-driven mitochondrial dysfunction and immune regulation may cooperate to promote BRCA progression and therapeutic resistance.

Several limitations should be acknowledged. First, the drug sensitivity data derived from public databases, such as the GDSC database, should be validated in cell culture experiments to confirm the relationship between LARP1 expression and drug response in breast cancer. These database-derived findings need to be experimentally verified in our *in vitro* models to ensure their relevance to specific subtypes of breast cancer. Additionally, while co-expressed genes were used for pathway enrichment analysis, future studies should demonstrate the effects of these genes in response to LARP1 knockdown in cells. Second, the genomic alteration analysis of LARP1 in breast cancer is constrained by the relatively low mutation burden in TCGA-BRCA; therefore, mutation-based inferences (including survival differences) should be interpreted cautiously. We present the mutation panel primarily as descriptive context rather than definitive functional evidence. To avoid over-interpretation from small BRCA event counts, we also summarized the pan-cancer mutation landscape to provide a more robust overview of alteration types and their distribution along the protein. Third, functional validation was performed using only two breast cancer cell lines (MDA-MB-231 and SK-BR-3), which may not fully capture the molecular heterogeneity of breast cancer. Future studies should extend these findings to additional subtypes and patient-derived models. Furthermore, our direct co-culture model does not distinguish between contact-dependent tumor-macrophage interactions and effects mediated by tumor-secreted soluble factors. Therefore, the macrophage polarization changes reflect an integrated tumor-immune interface. Future studies using Transwell systems, conditioned-medium assays, and pathway/cytokine blocking experiments will be required to delineate the relative contributions of these mechanisms.

## Conclusion

*LARP1* contributes to BRCA progression by regulating cell cycle-related signaling pathways, disrupting mitochondrial homeostasis, and modulating immune molecules. These results indicate that *LARP1* functions as an oncogene in BRCA and may serve as a valuable biomarker for diagnosis, prognosis, and characterization of the tumor immune microenvironment.

## Data Availability

The original contributions presented in the study are included in the article/**Supplementary Material**. Further inquiries can be directed to the corresponding author.
