# Supplementary figures and images for: The role of LARP1 in breast cancer progression: from prognosis to immune microenvironment remodeling
Source: Front Endocrinol (Lausanne). 2026 Jun 16;17:1764944. doi: 10.3389/fendo.2026.1764944 (PMC13314501; doi:10.3389/fendo.2026.1764944)

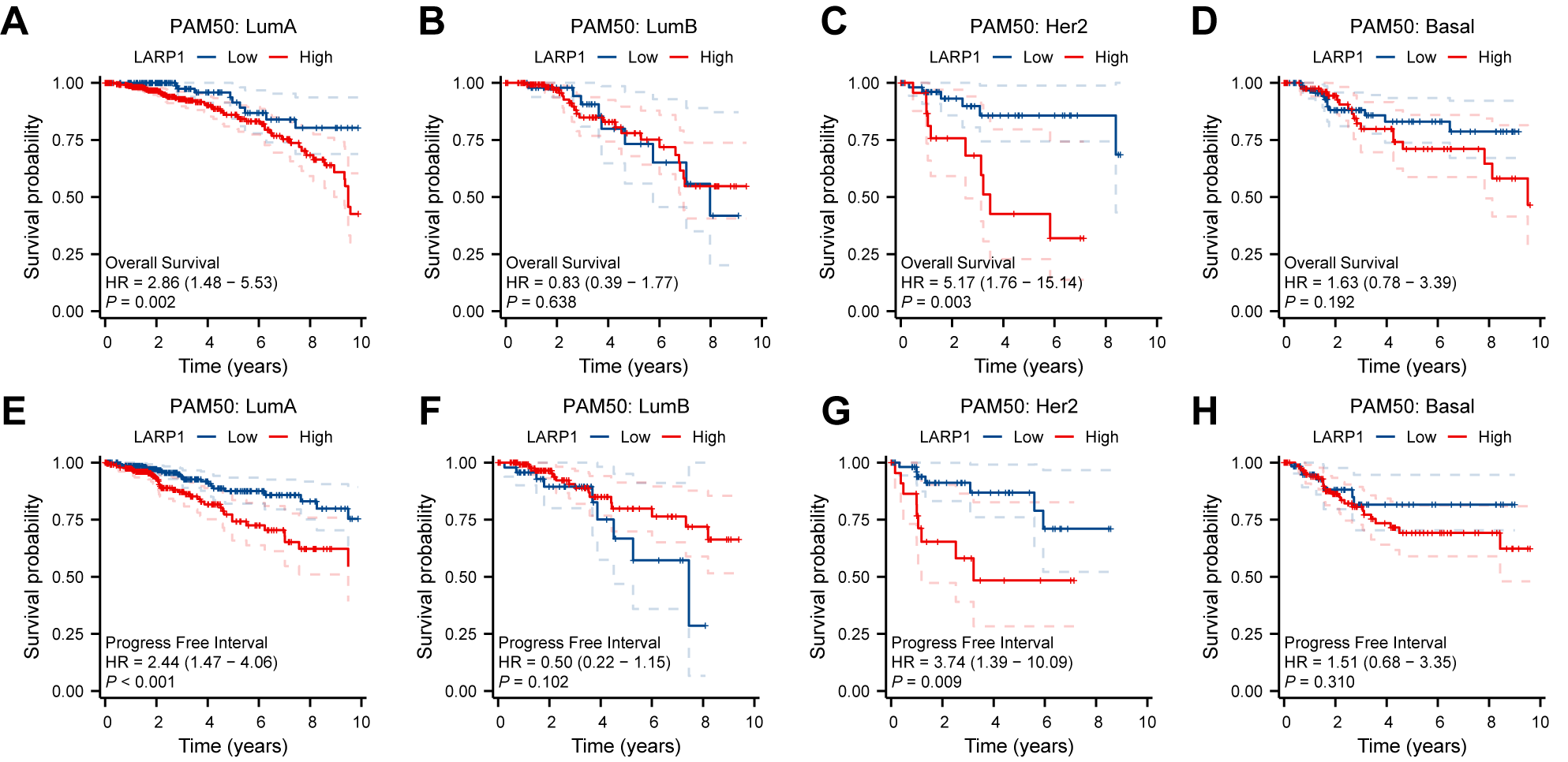

Supplement: Supplementary file 1 [file Image1.tif]

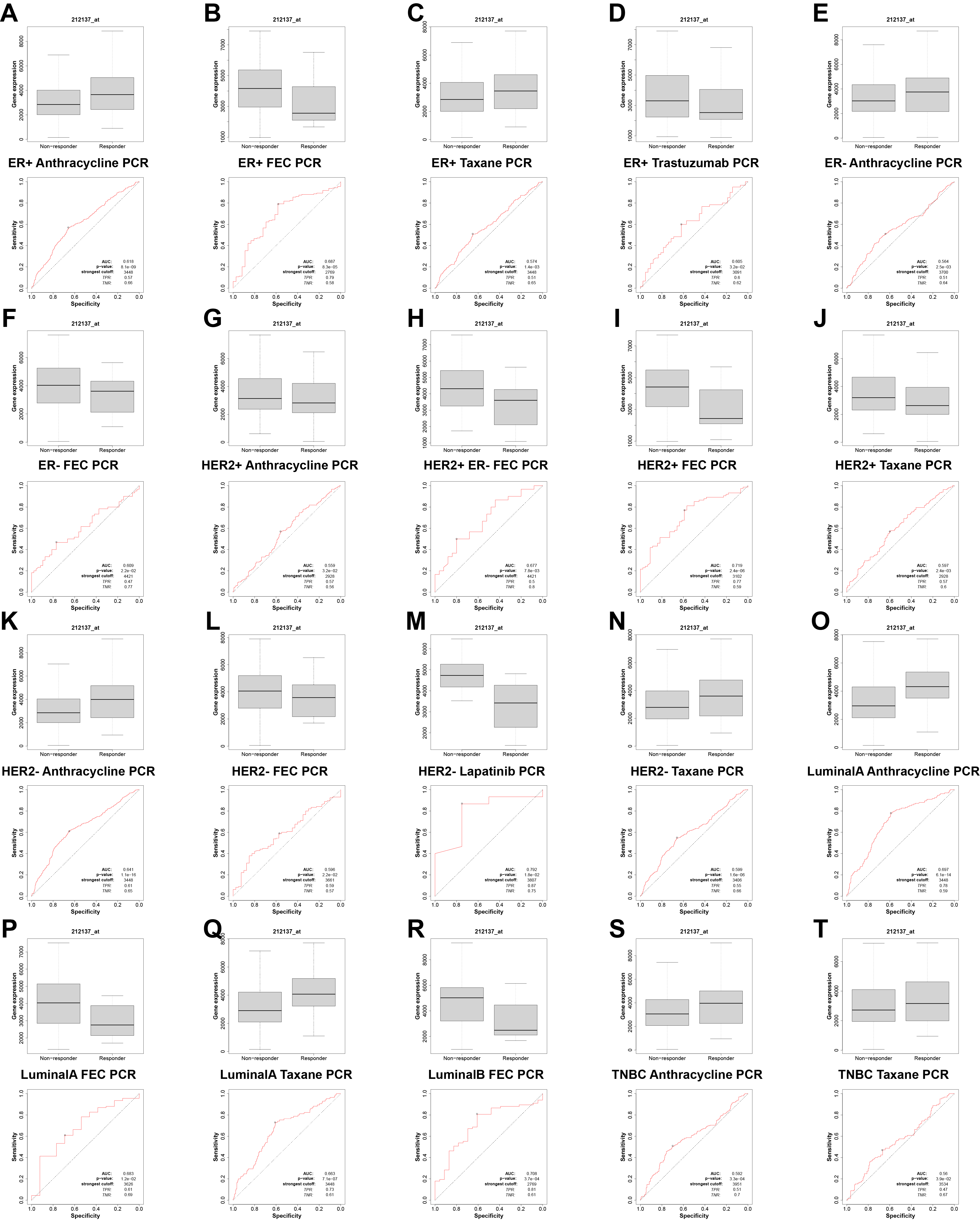

Supplement: Supplementary file 2 [file Image2.tif]

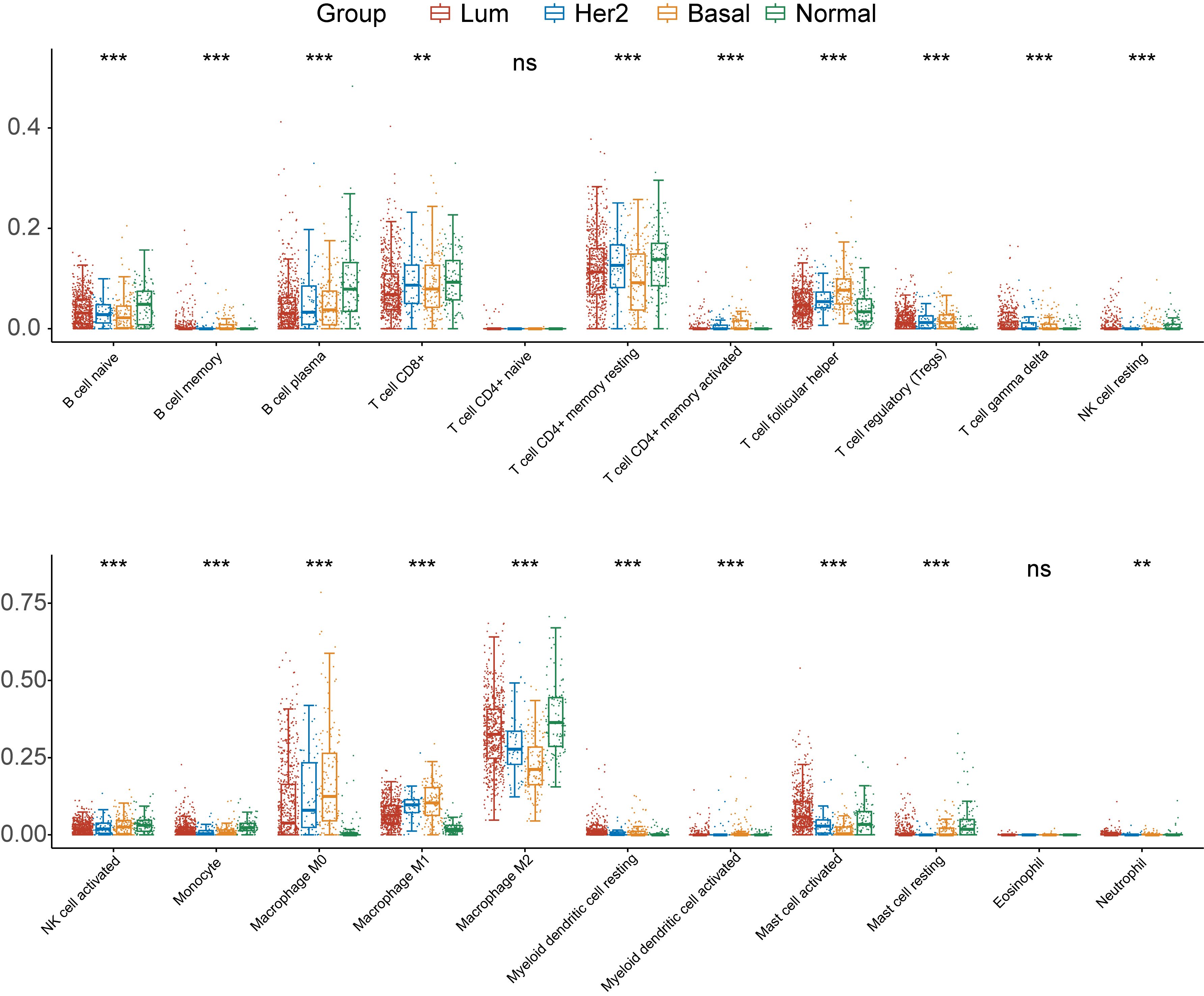

Supplement: Supplementary file 3 [file Image3.tif]
